# Postoperative outcomes of aspirin in microvascular free tissue transfer surgery—A systematic review and meta-analysis

**DOI:** 10.1016/j.jpra.2023.11.003

**Published:** 2023-11-10

**Authors:** Sahal A. Khan, Ramah K. Tayeb

**Affiliations:** a Department of Plastic and Reconstructive Surgery, Dijon Hospital, France; b King Abdulaziz University, Jeddah, Kingdom of Saudi Arabia

**Keywords:** Complication, Survival, Free flaps, Microvascular, Aspirin, Postoperative

## Abstract

Microvascular free tissue transfer surgery is a frequently used technique for head and neck reconstruction involving anticoagulants, and the present study aimed to analyse the postoperative outcomes of aspirin use in conjunction with this procedure. We searched databases for articles published between 2007 and 2022 on microvascular free tissue transfer surgery using aspirin and assessed them for primary and secondary outcomes. Odds ratios (ORs) and 95 % confidence intervals (CIs) were determined through analyses, followed by constructing a forest plot for complication rates. A total of 617 articles were retrieved from the databases, including 14 original full-text articles. Overall complication rates ranged from 0.7–38 % (95 % CI, 17.85 ± 0.503 (±2.8 %) [17.347–18.353]), while flap survival rates ranged from 95–99.2 % (95 % CI, 96.28 ± 0.0956 (±0.10 %) [96.184–96.376]). Two studies reported similar complication rates of 38 %, the highest among all reported studies. The ORs between the studies for the complications and flap survival rates were 2.614 and 0.722, respectively. Although the complication rates associated with aspirin use were not significantly high among the studies, they cannot be ignored. Flap survival rates were independent of the dose and type of anticoagulants used during surgery.

## Introduction

Microvascular free tissue transfer surgery is a reconstructive technique involving removing healthy tissue from one area of the body where there is an abundance or dispensability and transplanting it to another area of the body where a deficit exists generally after trauma, surgery for cancer, or chronic infection. Microvascular surgery is a type of microsurgery that involves the repair and anastomosis of small blood vessels (diameter < 3 mm),^1^ whereas super microsurgery involves microvessels (diameter 0.3–0.8 mm).^2^ Recently, the safety and reliability have been reviewed for the combined performance of microvascular free tissue transfer surgery and mesh repair for abdominal wall restoration.^3^ The overall success rate of microvascular free tissue transfer surgery is 90–98 %.^4^

Free flap failures still occur despite the documented safety and efficacy of microvascular free tissue transfer. However, major bleeding or thrombosis at the venous or arterial anastomosis is the main cause of concern after this type of surgery. Over the past few decades, different anticoagulation protocols have been developed to reduce the likelihood of these complications, albeit with varying degrees of success. To this end, prophylactic anticoagulant medications are frequently administered to patients undergoing free flap reconstruction. According to 96 % of the reconstructive surgeons who responded to a recent survey, patients undergoing free flap reconstruction are often prescribed an anticoagulant regimen that includes heparin, low-molecular-weight dextran, and/or aspirin, among others.^5^

Aspirin, a salicylate, is a well-established anticoagulant used as a pain reliever and anti-inflammatory drug, with an origin dating back several millennia. Due to its antiplatelet properties, aspirin is also used to treat coronary artery disease, stroke, and placental vascular diseases. The incidence of preeclampsia, intrauterine growth restriction, and foetal death due to vascular disorders has been reduced by the widespread use of aspirin dating back to the 1980s. A key component of platelet function, thromboxane A2, is irreversibly inhibited by aspirin. Due to its effect on platelet function, aspirin is widely used to prevent and treat acute myocardial infarction. As with any surgery, microvascular free tissue transfer still carries a significant risk of thrombosis.^6^ The present systematic review and meta-analysis, therefore, aimed to analyse the postoperative complication rates of microvascular free tissue combined with an aspirin regiment, with overall complication rates as the primary outcome and flap survival and failure rates as the secondary outcomes.

## Materials and methods

### Study design

The present systematic review and meta-analysis were performed following the guidelines of the Preferred Reporting Items for Systematic Reviews and Meta-Analyses (PRISMA) statement, using the population, intervention, control, and outcome (PICO) criteria. The PICO statement of the present study is as follows: P, patients who underwent a free tissue transfer procedure; I, use of aspirin as a prophylactic medication after free tissue transfer; C: comparison of free tissue transfer complication prevention with and without an aspirin regimen; O: what is the effect of using aspirin on flap loss after free tissue transfer? We aimed to analyse all published studies on aspirin use in microvascular free tissue transfer surgery and summarise the success and failure rates, biological events, complications, and potential future perspectives.

### Inclusion and exclusion criteria

Articles inclusion criteria were: (1) prospective and retrospective analyses; (2) studies involving patients who underwent free tissue transfer (free flaps); (3) studies using aspirin in any free tissue transfer for the prevention of flap loss; (4) studies published between 2007 and 2022 (15 years); and (5) original research articles with the full text in English. The exclusion criteria were as follows: (1) single case reports; (2) systematic reviews and meta-analyses; (3) studies published before 2007; (4) studies published in non-English languages; (5) studies with small sample sizes, < 5 patients; (6) studies without comparison groups; (7) studies that did not use aspirin; and (8) studies conducted on nonhuman subjects.

### Literature selection

#### Search strategy

Only full-text articles published in English were included in the present systematic review and meta-analysis. We searched the following databases for articles published between January 2007 and today (January 2023): PubMed, PubMed Central, Cochrane databases, Google Scholar, MEDLINE, EMBASE, SCOPUS databases, and Web of Sciences. The search terms and strings used for the analysis were (“aspirin” or “acetylsalicylic acid”) AND (“microvascular free tissue transfer” or “free tissue transfer” or “free flap” or “flap loss”). To find and include more eligible studies, a reference list was manually compiled from the retrieved studies and related reviews, and the process was repeated until no further articles were identified.

#### Study selection and data extraction

Following the initial screening process, 2 researchers independently read the entire texts of the articles that met the inclusion criteria. A third researcher reviewed the articles only in the event of a disagreement between the 2 researchers. Relevant data, such as article type, first author name, year of publication, number of patients, age and sex distribution, type of surgical procedure, comparison of aspirin with other compounds, primary and secondary outcomes, associated complications, and study limitations, were extracted from each included article.

#### Literature quality evaluation – the risk of bias and meta-analysis

The quality of all the studies included in the present analysis was evaluated, and a risk of bias assessment was performed using the Robivis (ROB 2.0) assessment tool. The 2 researchers who independently read the included literature scored the selected articles using the tool mentioned earlier. In the event of a disagreement, a third researcher was selected to reach a consensus.

### Statistical analysis

We used IBM SPSS Statistics for Windows (version 26.0; IBM Corp., Armonk, NY, USA) for statistical analysis. The incidence rate (odds ratio [OR]) was calculated by analysing dichotomous variables, and the interval estimation was expressed using a 95 % confidence interval (CI), with P < 0.05 indicating statistically significant differences. The size of the effect was calculated as the standardised mean deviation. We calculated the OR for complication rates and subsequently created a forest plot, using Microsoft Excel, to represent the overall complication rates associated with the use of an aspirin in conjunction with microvascular free tissue transfer surgery.

## Results

### Literature selection

**Figure 1:**
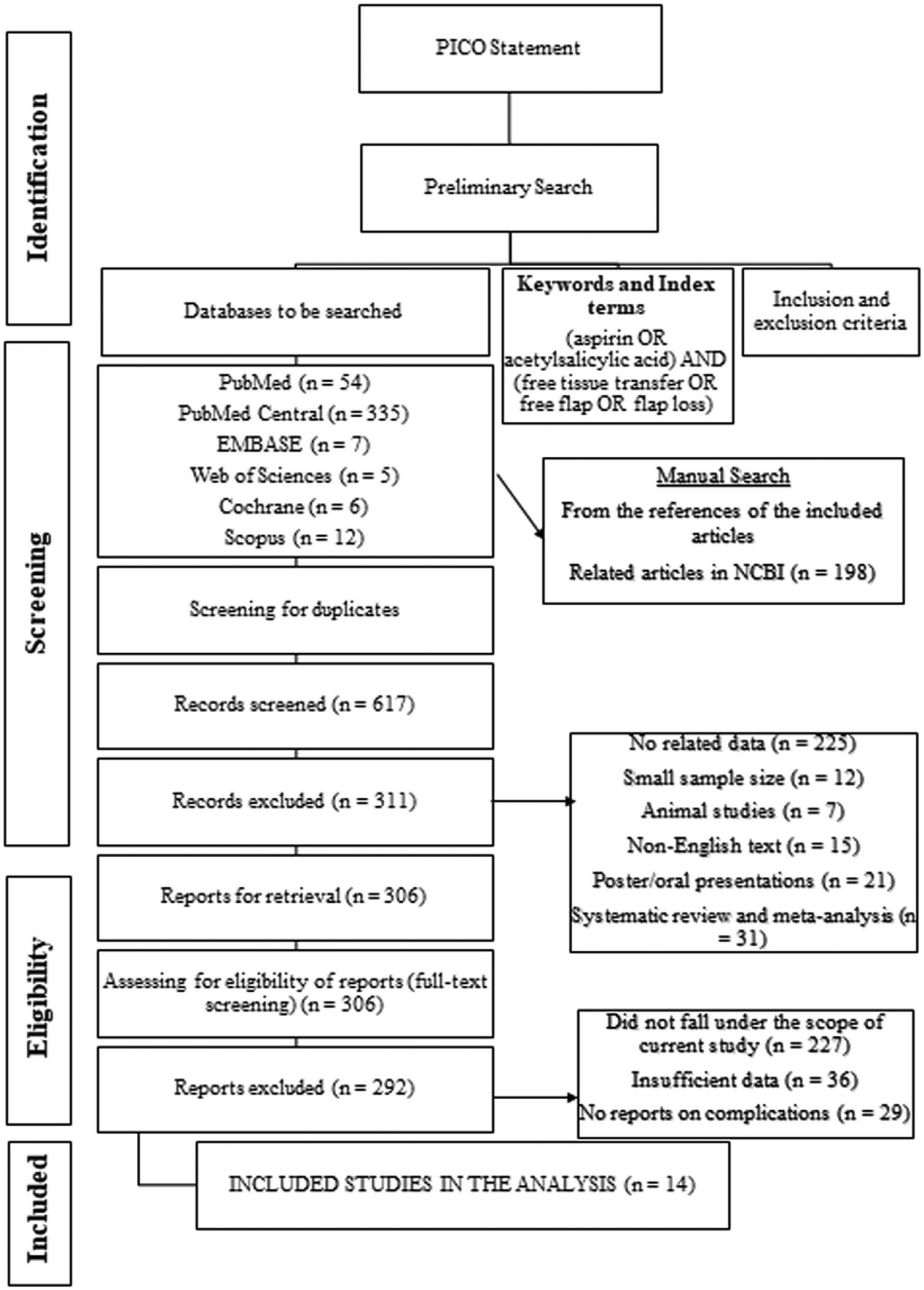
Preferred reporting items for systematic reviews and meta-analyses (PRISMA) chart for the study design.

A total of 617 articles were retrieved from PubMed, PubMed Central, EMBASE, Web of Science, Cochrane, MEDLINE, and SCOPUS databases, which were manually reviewed for inclusion/exclusion criteria. After the initial screening, 311 articles were excluded because they had unrelated data, small sample sizes, nonhuman participants, non-English abstracts or texts, poster and oral presentations, and/or systematic reviews and meta-analyses. After the final screening, a total of 14 full-text original articles that met all the inclusion criteria were included in the present study. The PRISMA chart for the present study, which provides detailed data on the literature collection and screening, is shown in Figure 1. Among the 14 included articles, 7 were retrospective analyses, 2 were prospective studies, 2 were interventional and therapeutic studies, and 3 were comparative studies, randomised control trials, or case series.

### Characteristics of the literature

The 14 studies in the present research encompassed 6,738 patients who underwent microvascular free tissue transfer surgery for face, head, and/or neck, breast, trunk, pelvis, and/or lower extremity, and/or post-skin cancer reconstruction. The age of the patients ranged from 31–76 years, with a male-to-female ratio of 53:47 (2 studies did not report sex data), and 12 of the 14 included studies reported thrombosis as their primary postoperative complication, indicating the significant occurrence of this complication, whereas venous congestion was only reported in the 2 remaining studies. Of the 14 studies, 5 did not disclose the concentration or dosage of aspirin; however, the other 9 studies reported that the dosage of aspirin ranged from 81–100 mg/day, with a median dose of 90.5 mg/day and an administration period of 5–14 days. The complication rate of microvascular free tissue transfer surgery with an aspirin regimen ranged from 0.7–38 %, with a significantly higher mean of 12.5 %. Supplementary Table S1 shows the demographic and baseline characteristics of all patients in the present study.

### Literature quality evaluation

#### Risk of bias

**Figure 2:**
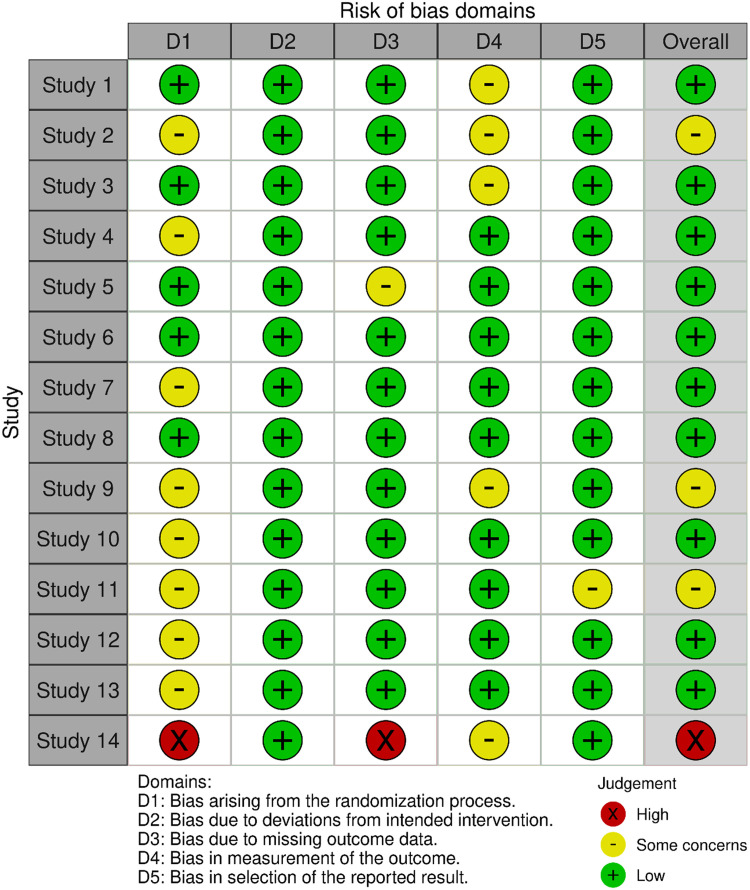
Risk of assessment plot.

**Figure 3:**
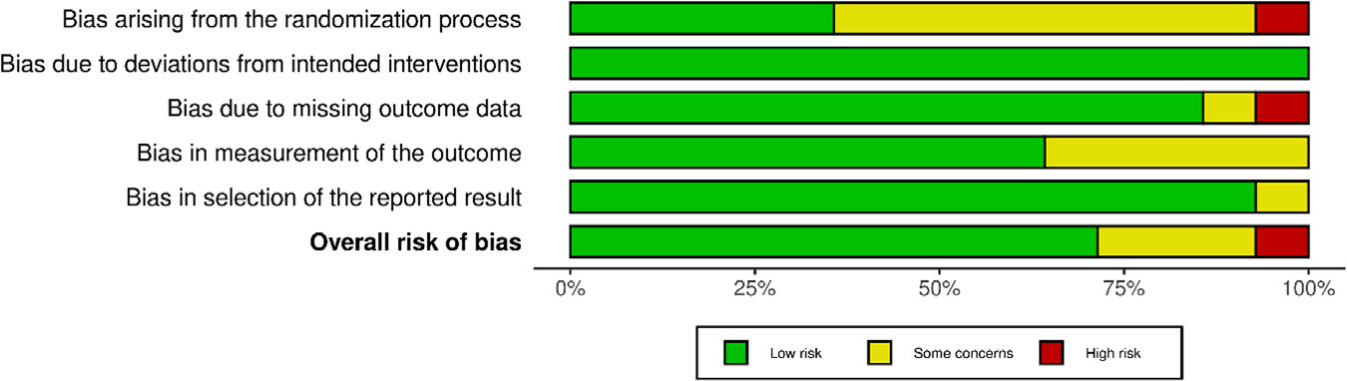
Risk of assessment summary.

Figures 2 and 3 show the variables included in the risk of bias assessment plot and summary, respectively. Of the 14 included studies, only 1 had a high risk of bias, whereas 3 had some concerns and 10 had a low risk.

#### Meta-analysis

The overall complication rates of microvascular free tissue transfer surgery ranged from 0.7–38 % (95 % CI, 17.85 ± 0.503 (±2.8 %) [17.347–18.353]), whereas overall flap survival rates ranged from 95–99.2 % (95 % CI, 96.28 ± 0.0956 (±0.10 %) [96.184–96.376]). Two of the studies reported similar complication rates—38 %, the highest among all reported studies. The ORs between the studies for overall complications and flap survival rates were 2.614 and 0.722, respectively.

**Figure 4:**
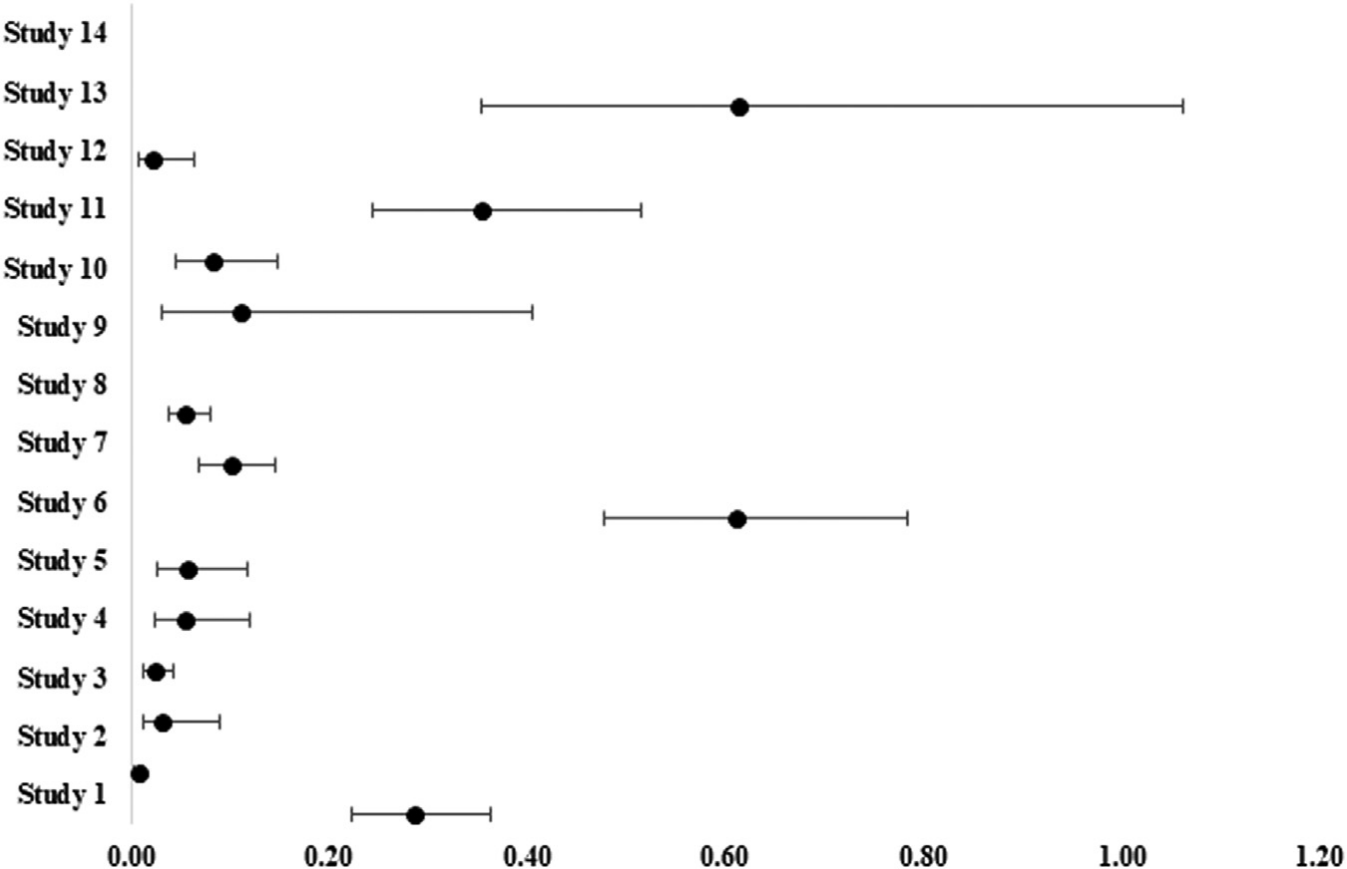
Forest plot showing the complication rates associated with aspirin use in microvascular free tissue transfer surgery among the included studies.

Supplementary Table S2 shows the basic details of the studies and the number of patients included in the present systematic review and meta-analysis. Table 1 shows the characteristics of the interventions and their associated complications.^7^, ^8^, ^9^, ^10^, ^11^, ^12^, ^13^, ^14^, ^15^, ^16^, ^17^, ^18^, ^19^, ^20^ Figure 4 shows a forest plot representing the complication rates associated with aspirin use in microvascular free tissue transfer surgery, whereas the data used to construct the forest plots are presented in Supplementary Table S3.

**Table 1:** Study characteristics, outcomes, and associated complications.

S. No.	Study	Type of surgical procedure	Anticoagulant used	Study outcomes		Associated complications
				Primary outcomes	Secondary outcomes	
1	Ashjian et al.,^7^	Free flap surgery for oncologic defects	Aspirin vs. heparin	No significant difference in bleeding, thromboembolism and flap loss between aspirin and heparin groups		Total and partial flap loss, infection, seroma, hematoma, bleeding, and pulmonary embolus death. Overall complication rate: 22.3 %
2	Dixon et al.,^8^	Flap repair after skin cancer	Aspirin vs. warfarin	Bleeding complications were lower in aspirin group		Overall complication rate: 0.7 %; In warfarin group: 2.5 %
3	Chernichenko et al.,^9^	Microsurgical free flap reconstruction	Aspirin (81 mg/dL for 2 weeks)	Flap survival rate: 97.6 %; microvascular free flap reconstruction success rate: 95–97 %		One flap failure, arterial and venous thrombosis, venous congestion. Overall complication rate: 3.2 %
4	Chen et al.,^10^	Free flap surgery for head and neck reconstruction	Aspirin and heparin	Combination of heparin and aspirin showed lower venous thrombus embolism rates		Overall complication rate: 2.35 %
5	Reiter et al.,^11^	Free flap surgery for neck and head reconstruction	Subcutaneous low-molecular weight enoxaparin	Flap survival rate: 97.1 %	Surgical intervention was required in 2.9 % of patients only	Total and partial flap failures due to venous thrombosis; salivary fistula; and postoperative hematoma. Overall complication rate: 15.3 %
6	Okochi et al.,^12^	Free flap surgery for neck and head reconstruction	Aspirin dose (100 mg/day) vs. non-antithrombotic group	Rate of flap necrosis was higher in aspirin (antithrombotic) group.	No difference in operation time, and intra- and postoperative bleeding between 2 groups	Total flap loss, acute myocardial infarction, cerebral infarction and non-thrombotic complications. Overall complication rate: 5.3 %
7	Lighthall et al.,^13^	Free flap tissue transfer	Aspirin vs. heparin	Complications were higher in aspirin group; flap failure, re-exploration, and salvage had no significant difference between aspirin and heparin groups	Postoperative revisions were more in the aspirin group	Total flap loss, infection, fistula, hematoma or haemorrhage, seroma, death and other medical complications. Overall complication rate: 38 %
8	Senchenkov et al.,^14^	Free flap surgery for neck and head, breast, extremity, trunk, or pelvis reconstruction	Aspirin, clopidogrel, heparin, tissue plasminogen activator, and Dextran-40	Success rate per flap: 99.5 %; success rate per patient: 99.2 %; overall microvascular salvage rate: 89 %; flap re-exploration rate: 92 %	Aspirin with other anticoagulants - combined infusion: no hematoma occurrence (except 2, which was evaluated surgically)	Two flaps lost (arterial thrombosis); Thrombotic events (n = 15), non-thrombotic insufficiencies, hematomas, negative exploration. Overall complication rate: 9 %
9	Eichhorn et al.,^15^	Local flap surgery or skin grafts	Clopidogrel vs. aspirin + clopidogrel	Complication rate was lower in combined group		Bleeding complication rate: 5.3 % vs. 4.2 %
10	Karimi et al.,^16^	Free flap surgery for head and neck reconstruction	Aspirin and enoxaparin	Flap success rate: 100 % Need for re-exploration: 10 %	No complete necrosis or loss of flap	Overall complication rate: 10 %
11	Karamanos et al.,^17^	Free tissue transfer of the extremities	Aspirin (81 mg vs. 325 mg)	Baby dose daily aspirin showed lower incidence of reoperation for arterial anastomosis	No difference in complete and partial flap loss, wound dehiscence, or infection between groups	Overall complication rate: 7.6 %
12	Mishu et al.,^18^	Lower extremity free tissue transfer	Aspirin and clopidogrel	Overall flap success rate: 90–95 %	Dual antiplatelet therapy withholding on free flap transfer did not influence intraoperative transfusion	Cardiac events. Overall complication rate: 26.4 %
13	Rothweiler et al.,^19^	Free flap reconstruction for malignant neoplasms	Aspirin and heparin	Long-term aspirin (>72 hr) increases complication rate; short term aspirin is beneficial. Heparin reduces flap loss but increases complication when administered in higher dose	No association between ischaemia time and complications was found	Thrombosis and major postoperative bleeding. Overall complication rate: 2.5 %
14	Taylor et al.,^20^	Head and neck free flap surgery	Aspirin (81 mg/day) and enoxaparin	Flap survival rate: 95 %; flap failures: 5 %		Venous congestion. Overall surgical complication rate: 38 %

## Discussion

Microvascular free tissue transfer surgery often involves using anticoagulants as postoperative medications to prevent possible complications and flap failures. Microvascular free tissue transfer is the most widely used head and neck reconstruction technique. Despite its rarity, flap failure can occur, and when it does, it has a profound physical and emotional impact on patients’ lives. Furthermore, head and neck complications after free tissue transfer are common,^21^ although the incidence of complications associated with anticoagulant use remains to be clarified. Studies recommending a standard dosage of anticoagulants for microvascular free tissue transfer surgery are still lacking, although heparin, aspirin, and dextran are the most commonly used anticoagulants. The results of the present systematic review and meta-analysis of the postoperative complications associated with aspirin use in patients undergoing microvascular free tissue transfer surgery showed that the overall complication rates were significant. Although the complication rate varied from 0.7–38 %, the mean rate was considerably higher, at 12.5 %.

One systematic analysis reported that aspirin had the lowest rate of flap failure compared with dextran and heparin^14^; however, in the present study, aspirin combined with dextran had a positive impact with a lower complication rate. A study by Swartz et al.^22^ reported that flap survival was negatively associated with the type of anticoagulant used in conjunction with the microvascular free tissue transfer surgery; however, the flap survival rates were found to be higher in all of the included studies, irrespective of the anticoagulant used.

It has been reported that patients undergoing head and neck reconstruction using a concise anticoagulation regimen of aspirin and subcutaneous heparin appear to have a free flap survival rate that is comparable to patients using other anticoagulants.^5^ Hwang et al.^23^ have reported that elderly patients undergoing microvascular free tissue transfer surgery for head and neck reconstruction experience higher complications related to memory, such as dementia. Additionally, aspirin may lead to gastrointestinal tract bleeding.^24^ Disa et al.^25^ compared postoperative anticoagulant regimens, such as aspirin and dextran, and reported that dextran was associated with significantly more systemic complications than aspirin and that there was no significant evidence that dextran was more effective than aspirin for the prevention of thromboses.

Thrombosis after microvascular free tissue transfer surgery continues to be a problem, which can ultimately result in flap failure. In most cases of flap failure, the thrombosis occurred before the third postoperative day. Aspirin-related haemorrhagic complications most commonly manifest in the digestive tract or skin but occasionally do occur in the brain. Major bleeding, including gastrointestinal bleeding and intracranial haemorrhage, was reported to be similar between high- (300–325 mg daily) and low- (75–100 mg daily) dose aspirin in the CURRENT-OASIS 7 study,^26^ whereas Raggio et al.^27^ suggested that the use of aspirin is significantly associated with the formation of hematomas. In the present systematic review and meta-analysis, hematomas were reported in 4 studies, similar to the result of previous studies. Out of the 14 studies included in the present meta-analysis, flap survival rate after aspirin usage was 100 % in only one study.^16^ The remaining 13 studies, however, showed a flap survival rate > 90 %, which is significant. Additionally, the aspirin dosage did not appear to affect the primary or secondary outcomes in the included studies.

The fact that we only included studies involving aspirin with sample sizes ≥ 30 is one of the strengths of the present study, which had a few limitations. First, the sample size varied from 30–2394, which could have induced bias in the assessment. Second, the heterogeneity of the included studies could not be defined because the results did not vary significantly.

Based on our findings, we hypothesise that the complication rates associated with aspirin use after microvascular free tissue transfer surgery are not statistically significant, although they cannot be ignored. The flap survival rates were independent of the dose and type of anticoagulant(s) used during and after surgery. Further large-scale studies with similar patient distributions are required to confirm the present study's findings. Several future studies should be conducted based on the discussed limitations to confirm the findings of this research. First, a meta-analysis of studies with larger sample sizes should be conducted to determine the complication rates associated with aspirin use after microvascular free tissue transfer surgery. Second, a systematic review of studies with a standardised protocol should be conducted to assess the studies' heterogeneity and determine whether the results are consistent across the studies. Third, a randomised controlled trial comparing the use of aspirin versus placebo in microvascular free tissue transfer surgery should be conducted to determine whether there is a significant difference in the overall complication rates. Fourth, a retrospective cohort study should be conducted to compare the overall complication and flap survival rates in patients who received different doses and types of anticoagulants during and after microvascular free tissue transfer surgery. Finally, a prospective cohort study with a large sample size should be conducted to confirm the findings of the present study and to assess the long-term outcomes of microvascular free tissue transfer surgery. Overall, these studies could provide a more comprehensive understanding of the outcomes associated with microvascular free tissue transfer surgery and help address the limitations of the present study.

## CRediT authorship contribution statement

**Sahal A. Khan:** Conceptualization, Writing – original draft, Formal analysis, Writing – review & editing. **Ramah K. Tayeb:** Methodology, Software, Writing – review & editing, Conceptualization.

## Appendix Supplementary materials

Image, application 1
