# Supplementary material for: Postoperative outcomes of aspirin in microvascular free tissue transfer surgery—A systematic review and meta-analysis
Source: JPRAS Open. 2023 Nov 10;39:49–59. doi: 10.1016/j.jpra.2023.11.003 (PMC10755481; doi:10.1016/j.jpra.2023.11.003)
Supplement: Supplementary file 1 [file mmc1.docx]

**Supplementary Table S1. Demographic and baseline characteristics of the patients included in the study**

| ***S. No*** | ***Mean age (years)*** | ***Male*** | ***Female*** | ***Reconstruction site*** | ***Reference*** |
| --- | --- | --- | --- | --- | --- |
|  | 55.5 | 203 | 267 | Head and neck, trunk and breast, upper and lower extremities | Ashjian et al., 2007^7^ |
|  | 64 | 1324 | 1070 | Skin | Dixon et al., 2007^8^ |
|  | 66 | 89 | 35 | Anterior  skull base, larynx, oral cavity, oropharynx, temporal  bone, scalp, skin resurfacing, orbit, cervical esophagus,  mandible, and maxilla | Chernichenko et al., 2008^9^ |
|  | 58.1 | 773 | 818 | Head and neck | Chen et al., 2008^10^ |
|  | 51.8 | 83 | 45 | Head and neck | Reiter et al., 2012^11^ |
|  | 69 | NR | NR | Head and neck | Okochi et al., 2012^12^ |
|  | 64.3 | 193 | 197 | Radial forearm, anterolateral, thigh, fibula,  Latissimus dorsi,  rectus, jejunum or ileocolic, scapula, and ulnar | Lighthall et al., 2013^13^ |
|  | 51 | 47 | 208 | Neck and head, breast, extremity, trunk, or pelvis | Senchenkov et al., 2015^14^ |
|  | 73 ± 7.9 | 356 | 294 | Skin grafts in cheek, forehead, and ear | Eichhorn et al., 2015^15^ |
|  | 45.0 | 21 | 9 | Head and neck | Karimi et al., 2016^16^ |
|  | 31 | 125 | 58 | Head and neck | Karamanos et al., 2022^17^ |
|  | 56.5 | NR | NR | Lower extremity | Mishu et al., 2022^18^ |
|  | 63.4 | 105 | 73 | Malignant neoplasms | Rothweiler et al., 2022^19^ |
|  | 58.8 | 60 | 19 | Head and neck | Taylor et al., 2021^20^ |

NR, not reported

**Supplementary Table S2. Basic Details of the Included Studies**

| ***S. No.*** | ***Publication year*** | ***Article type*** | ***Period of study*** | ***Number of patients/ free flaps*** |
| --- | --- | --- | --- | --- |
|  |  |  |  |  |
| 1 | Ashjian et al., 2007^7^ | Research Article – Prospective analysis | 2002–2005 | 505 in 470 patients |
| 2 | Dixon et al., 2007^8^ | Research Article - Comparative Study | 2002–2006 | 2394 |
| 3 | Chernichenko et al., 2008^9^ | Research Article | 2001–2006 | 127 in 124 patients |
| 4 | Chen et al., 2008^10^ | Retrospective analysis | 1997–2006 | 1591 |
| 5 | Reiter et al., 2012^11^ | Retrospective analysis | 2007–2010 | 137 in 128 patients |
| 6 | Okochi et al., 2012^12^ | Research Article | 2001–2006 | 150 patients/ 9 cases in detail |
| 7 | Lighthall et al., 2013^13^ | Case series | 2006–2010 | 390 |
| 8 | Senchenkov et al., 2015^14^ | Retrospective analysis | 2006–2010 | 395 in 255 patients |
| 9 | Eichhorn et al., 2015^15^ | Retrospective Analysis | 2005–2015 | 650 |
| 10 | Karimi et al., 2016^16^ | Interventional study | 2013–2014 | 30 |
| 11 | Karamanos et al., 2022^17^ | Research Article | 2005–2019 | 183 |
| 12 | Mishu et al., 2022^18^ | Research Article | 2011–2019 | 195 |
| 13 | Rothweiler et al., 2022^19^ | Research Article | 2013–2018 | 178 |
| 14 | Taylor et al., 2021^20^ | Prospective Randomized Control Trial | NR | 79 |

| ***Study*** | ***Odds ratio*** | ***Lower Limit 95% CI*** | ***Upper Limit 95% CI*** |
| --- | --- | --- | --- |
| Study 1 | 0.28 | 0.22 | 0.36 |
| Study 2 | 0.01 | 0.00 | 0.01 |
| Study 3 | 0.03 | 0.01 | 0.09 |
| Study 4 | 0.02 | 0.01 | 0.04 |
| Study 5 | 0.05 | 0.02 | 0.12 |
| Study 6 | 0.06 | 0.03 | 0.12 |
| Study 7 | 0.61 | 0.48 | 0.78 |
| Study 8 | 0.10 | 0.07 | 0.15 |
| Study 9 | 0.06 | 0.04 | 0.08 |
| Study 10 | 0.11 | 0.03 | 0.41 |
| Study 11 | 0.08 | 0.05 | 0.15 |
| Study 12 | 0.35 | 0.24 | 0.52 |
| Study 13 | 0.02 | 0.01 | 0.06 |
| Study 14 | 0.61 | 0.35 | 1.06 |

**Supplementary Table S3: Values used for constructing a forest plot**
